# Sex differences in the association between cardiovascular risk factors and coronary artery calcification progression among individuals without coronary artery calcium

**DOI:** 10.1186/s13293-025-00802-8

**Published:** 2025-12-07

**Authors:** Jia-Jie Wang, Yian Yao, Guoli Sun, Zi Ye, Hungchen Lin, Chengxing Liu, Yan Lai, Ying Zhang, Zhichao Zheng, Xuebo Liu

**Affiliations:** 1 Department of Cardiology, Tongji Hospital, School of Medicine, Tongji University, No 389, Xincun Road, Putuo District, Shanghai, 200333 China; 2 Department of Cardiology, Guangdong Provincial People’s Hospital (Guangdong Academy of Medical Sciences), Southern Medical University, No. 106, Zhongshan 2nd Road, Guangzhou, 510080 Guangdong Province China

**Keywords:** Cardiovascular risk factors, Coronary artery calcium progression, Sex difference

## Abstract

**Background:**

Cardiovascular risk factors are determinants of coronary artery calcium (CAC) progression. However, whether the effect of cardiovascular risk factors on CAC progression among participants with CAC = 0 differs by sex remains unclear.

**Method:**

This study included 1815 participants 33–45 years of age from the Coronary Artery Risk Development in Young Adults study at baseline who had CAC measured both at baseline and five years later. CAC was measured using computed tomography. Risk factor measurements included body mass index (BMI), waist circumference, total cholesterol, triglycerides, glucose, systolic blood pressure (BP), low-density lipoprotein cholesterol, and smoking status.

**Results:**

CAC progression was significantly higher among men than women (2.25 (8.68) vs. 0.89 (6.7), *P* < 0.0001). In the restricted cubic spline models, the associations between systolic BP and CAC progression in women followed a nonlinear relationship. The slope for the regression of systolic BP on CAC progression in women was relatively flat until around 125 mmHg of systolic BP and then started to increase rapidly afterwards, with a β of 0.16. BMI was associated with CAC progression only in men. Similar associations were observed when replacing BMI with waist circumference. There were no sex differences in the associations between CAC progression and smoking.

**Conclusion:**

Systolic BP in women and BMI (and/or waist circumference) in men may have different contributions to CAC progression between the sexes among participants with CAC = 0. Our study provides evidence that understanding sex differences in cardiovascular risk factors is essential for implementing targeted interventions to prevent CAC progression.

**Supplementary Information:**

The online version contains supplementary material available at 10.1186/s13293-025-00802-8.

## Abstract (plain language summary)

Cardiovascular risk factors are determinants of coronary artery calcium (CAC) progression. However, whether the effect of cardiovascular risk factors on CAC progression among participants with normal CAC differs by sex remains unclear. This study included 1815 participants 33–45 years of age from the Coronary Artery Risk Development in Young Adults study at baseline who had CAC measured both at baseline and five years later. CAC was measured using computed tomography. Risk factor measurements included body mass index (BMI), waist circumference, total cholesterol, triglycerides, glucose, systolic blood pressure (BP), low-density lipoprotein cholesterol, and smoking status. CAC progression was significantly higher among men than women. Systolic BP was associated with CAC progression only in women, and BMI was associated with CAC progression only in men. Similar associations were observed when replacing BMI with waist circumference. There were no sex differences in the associations between CAC progression and smoking. Systolic BP in women and BMI (and/or waist circumference) in men may have different contributions to CAC progression between the sexes among participants with normal CAC. Our study provides evidence that understanding sex differences in cardiovascular risk factors is essential for implementing targeted interventions to prevent CAC progression.

**Supplementary Information:**

The online version contains supplementary material available at 10.1186/s13293-025-00802-8.

## Highlights

We evaluate sex differences in the association between cardiovascular risk factors and CAC progression among participants with CAC=0.

The associations between systolic BP and CAC progression in women followed a nonlinear relationship.

BMI (and/or waist circumference) was associated with CAC progression only in men.

Systolic BP in women and BMI (and/or waist circumference) in men may have different contributions to CAC progression between the sexes among participants with CAC=0.

Our study provides evidence that understanding sex differences in cardiovascular risk factors is essential for implementing targeted interventions to prevent CAC progression.

**Supplementary Information:**

The online version contains supplementary material available at 10.1186/s13293-025-00802-8.

## Introduction

Subclinical arteriosclerosis, identified by the presence of coronary artery calcification (CAC), progresses over time and is associated with the development of subsequent coronary heart disease (CHD) events [1–4]. Conversely, absence of CAC (CAC = 0) is a powerful negative risk marker for the development of CHD [5, 6]. Considerable sex differences in CAC progression have been reported [7, 8]. An enhanced understanding of sex differences in CAC progression among participants with CAC = 0 is of clinical importance to elucidate the underlying pathophysiologic mechanisms and could ultimately lead to preventive strategies to reduce subsequent CHD events.

Cardiovascular risk factors, such as obesity, hypertension, diabetes, and smoking, are determinants of CAC progression [9–11]. Many studies have reported that cardiovascular risk factors confer differential excess risks on diseases such as myocardial infarction and coronary heart disease for women and men [12–15]. However, whether the effect of cardiovascular risk factors on CAC progression differs by sex remains unclear. Therefore, our study aimed to evaluate sex differences in the association between cardiovascular risk factors and CAC progression among participants with CAC = 0, using the data from the Coronary Artery Risk Development in Young Adults Study (CARDIA).

## Materials and methods

### Study population

The CARDIA study is a multicenter longitudinal cohort study that enrolled 5,115 healthy African American and white participants aged 18 to 30 years from 4 U.S. cities (Birmingham, Alabama; Oakland, California; Chicago, Illinois; and Minneapolis, Minnesota) in 1985 and 1986 [16, 17]. The study protocols were approved by the institutional review boards at each study site, and written informed consent was obtained from all participants.

A total of 5113 participants aged 33–45 attended the year 15 exam in 2000–2001. CARDIA year 15 was considered the baseline. Computed tomography (CT) was performed to measure CAC at year 15 and year 20 exam [18]. Of the 5,113 who attended the year 15 exam, 2277 were excluded for not undergoing electron beam cardiac CT during the year 15 and the year 20 exam. For the purpose of the present analyses, we further excluded those with missing data on body mass index (BMI), total cholesterol, triglyceride, glucose, blood pressure (BP), and low-density lipoprotein cholesterol (LDL-C) at baseline (*n* = 91), or those with missing data on CAC by CT scans at the year 15 and the year 20 exam (*n* = 330), or those with CAC >0 at year 15 exam (*n* = 221), or those who used antihypertensive medications or lipid-lowering medication or hypoglycemic medications (*n* = 172), or those who have a history of cardiovascular disease (*n* = 207). The final sample for this analysis consisted of 1815 participants (Figure S1 in Supplementary Materials).

### Cardiovascular risk factors and other covariates

At baseline, following standard CARDIA protocols, sex, race, age, smoking status, history of cardiovascular disease, and use of medications were self-reported [17]. Height, weight, and waist circumference were measured by trained personnel. BMI was calculated by dividing weight in kilograms by height in meters squared (kg/m^2^). BP was measured three times by a trained technician on the right arm with the participant seated after a 5-minute rest using a random-zero sphygmomanometer, and the average of the last two measurements was used. Plasma concentrations of total cholesterol, triglycerides, and LDL-C were determined using enzymatic procedures or estimated by the Friedewald equation, and serum fasting glucose was measured using hexokinase coupled to glucose-6-phosphate dehydrogenase, just as previously described [19, 20].

### Study outcome

CAC was assessed at the year 15 and the year 20 exam using computed tomography (CT) via multidetector scanners [18]. To measure CAC, we obtained contiguous 2.5–3 mm-thick transverse images from the root of the aorta to the apex of the heart. Images collected at each center were then transmitted electronically to the CARDIA Reading Center. Total CAC scores were calculated by image analysts blinded to participant characteristics. The CAC were scored using the Agatston method, which was calculated for each calcified lesion [21]. CAC progression was calculated as the difference of logarithmic CAC scores at the year 15 and the year 20 exam to correct for skewness (log[CAC (year 20) + 1]). Incident CAC was defined as CAC >0 at year 20.

### Statistical analysis

Categorical variables were presented as counts and percentages (%). Continuous variables were described using the means and standard deviations. Pearson Chi-squared test for categorical variables and Mann–Whitney U test for continuous variables were used to compare baseline descriptive statistics between men and women. The linear regression models were used to assess the association between cardiovascular risk factors and CAC progression after checking linearity, independence, multicollinearity, normality, and homoscedasticity. Robust standard errors were calculated to correct for heteroscedasticity. We used linear regression models stratified by sex to investigate the primary aim of defining the independent predictors of changes in CAC progression by sex. Three models were examined for each sex. First, univariate models for each risk factor were examined. Second, each cardiovascular risk factor was examined using minimally adjusted models, which were all adjusted for age and race. Third, multivariable models including all risk factors were constructed. To examine whether sex modifies the associations between risk factors and CAC progression, we tested sex risk factor interaction terms with multiplicative interaction analysis in the multivariable linear regression model, which included all risk factors. These analyses were performed separately for each risk factor. For potential non-linear relations, we used restricted cubic spline (RCS) to model the association between risk factors and CAC progression. The analysis was repeated, replacing BMI with waist circumference. In the sensitivity analysis, diastolic BP was additionally adjusted. Logistic regression analyses were used to examine the odds ratio and 95% CI for the incident CAC. All the statistical analyses were performed using SPSS version 26.0 and R version 4.2.0, and statistical significance was inferred as a *P* value of < 0.05.

## Result

### Clinical characteristic

The characteristics of study participants are shown in Table 1. The study cohort consisted of 1815 participants (784 men and 1031 women) with CAC = 0 at baseline with a mean (SD) age of 40.1 (3.57) years. Men had higher total Cholesterol, higher triglycerides, higher LDL-C, higher fasting glucose, and higher systolic BP but less likely to remained with CAC = 0 at year 20 than women. CAC progression was also significantly higher among men than women (2.25 (8.68) vs. 0.89 (6.7), *P* < 0.0001).

**Table 1:** Baseline characteristics of CARDIA participants stratified by sex

Characteristics	Total (*n* = 1815)	Men (*n* = 784)	Women (*n* = 1031)	*P*-value for Men vs. Women
Age, Mean (SD), years	40.1 (3.57)	39.91 (3.51)	40.24 (3.62)	0.037
Race				0.166
Black (%)	765 (42.15)	316 (40.31)	449 (43.55)	
White (%)	1050 (57.85)	468 (59.69)	582 (56.45)	
Body mass index, Mean (SD), kg/m^2^	27.89 (5.78)	27.5 (4.22)	28.19 (6.71)	0.582
Waist circumference, Mean (SD), cm	87.27 (12.96)	91.99 (10.53)	83.68 (13.49)	< 0.0001
Total Cholesterol, Mean (SD), mg/dL	183.36 (32.97)	185.82 (34.91)	181.49 (31.3)	0.011
Triglycerides, Mean (SD), mg/dl	94.33 (56.41)	109.34 (65.61)	82.93 (45.07)	< 0.0001
LDL-C, Mean (SD), mg/dl	112.38 (30.44)	117.72 (31.93)	108.32 (28.61)	< 0.0001
Glucose, Mean (SD), mg/dL	84.12 (14.42)	86.64 (15.03)	82.2 (13.64)	< 0.0001
Systolic blood pressure, Mean (SD), mmHg	111.07 (13.17)	113.51 (11.97)	109.22 (13.73)	< 0.0001
Smoking (%)	346 (19.06)	161 (20.54)	185 (17.94)	0.164
CAC = 0 at year 20 (%)	1635 (90.08)	665 (84.82)	970 (94.28)	< 0.0001
CAC change, Mean (SD)	1.47 (7.64)	2.25 (8.68)	0.89 (6.7)	< 0.0001

CAC, coronary artery calcification

CARDIA, Coronary Artery Risk Development in Young Adults

LDL-C, low-density lipoprotein cholesterol

### Sex differences in CAC progression

In the univariate model, BMI, total cholesterol, triglycerides, LDL-C, fasting glucose, and systolic BP in men were associated with CAC progression (Table S1 in Supplementary Materials). In women, only smoking was significantly associated with CAC progression. In the separate models adjusted for age and race, the results remained essentially similar (Table S1 in Supplementary Materials).

Multivariable linear regression models stratified by sex and *P* for sex risk factor interaction terms are shown in Table 2. Smoking was a significant independent predictor of CAC progression in both men (β = 0.086, *P* = 0.026) and women (β = 0.11, *P* = 0.008) after adjusting for each of the other cardiovascular risk factors. There were no sex differences in the associations between CAC progression and smoking (*P* for interaction = 0.961). Total Cholesterol, triglycerides, LDL-C, fasting glucose, and systolic BP were not associated with CAC progression in both sexes. A significant interaction was observed between sexes in BMI and CAC progression (*P* for interaction = 0.002). BMI (β = 0.13, *P* = 0.004) in men was associated with CAC progression, whereas in women the association was non-significant (β = 0.038, *P* = 0.289).

**Table 2:** Multivariable linear regression models for associations between cardiovascular risk factors and CAC progression stratified by sex and *P* values for sex risk factor interaction terms

Risk variable	*P* for interaction	Men		Women	
		β	*P* value	β	*P* value
Age		0.14	< 0.0001	0.02	0.479
White		0.058	0.097	0.062	0.095
BMI	0.002	0.13	0.004	0.038	0.289
Total Cholesterol	0.005	0.037	0.736	-0.048	0.584
Triglycerides	0.001	0.019	0.682	-0.044	0.136
LDL-C	0.016	0.057	0.597	0.078	0.389
Fasting glucose	0.042	0.073	0.284	-0.001	0.969
Systolic BP	0.402	0.038	0.38	0.093	0.073
Smoking	0.961	0.086	0.026	0.11	0.008

Regression coefficients were calculated using robust standard errors

Model: Contained age, race, BMI, total cholesterol, triglycerides, systolic BP, LDL-C, fasting glucose, and smoking

BMI, body mass index;

BP, blood pressure;

CAC, coronary artery calcification;

LDL-C, low-density lipoprotein cholesterol

**Fig. 1:**
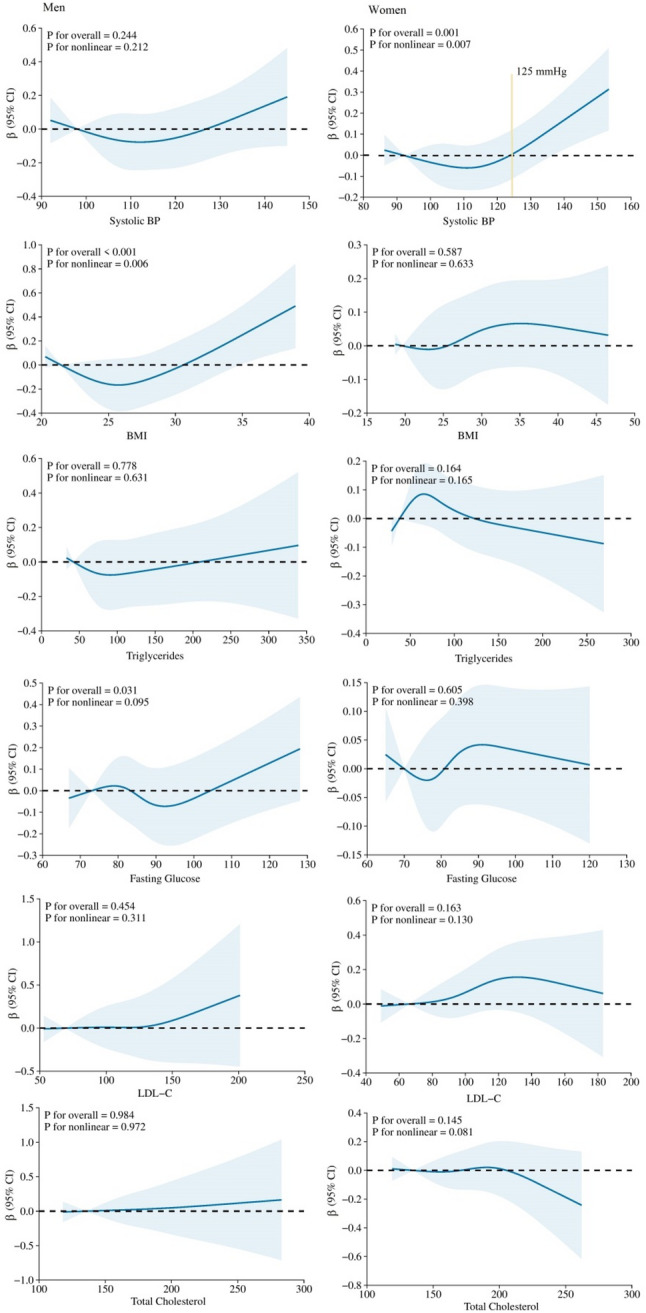
Multivariable adjusted spline of association between cardiovascular risk factors and CAC progression stratified by sex. Graphs show β for CAC progression according to cardiovascular risk factors. Model: Contained age, race, BMI, total cholesterol, triglycerides, systolic BP, LDL-C, fasting glucose, and smoking. The model was conducted with 4 knots at the 5th, 35th, 65th, 95th percentiles (reference is the 5th percentile). Solid lines indicate β, and shadow shape indicates 95% CIs. BMI, body mass index BP: blood pressure; CAC, coronary artery calcification; CI, confidence interval; LDL-C, low-density lipoprotein cholesterol

Sex-stratified RCS suggested nonlinear and sex-specific association patterns of systolic BP with CAC progression (Fig. 1). The slope for the regression of systolic BP on CAC progression in women was relatively flat until around 125 mmHg of systolic BP and then started to increase rapidly afterwards, with a β of 0.16 (Data not shown). We found no evidence of association between systolic BP and CAC progression in men. The associations between BMI and CAC progression in men were J-shaped. Conversely, BMI in women was not associated with CAC progression. With the use of < 30 kg/m^2^ (for BMI) and < 125 mmHg (for systolic BP) as references, Obesity in men (β = 0.13, *P* = 0.004) and elevated BP in women (β = 0.093, *P* = 0.033) were independently associated with CAC progression (Table 3). In addition, we found the associations of BMI and systolic BP with CAC progression also followed a nonlinear relationship in the entire population (Figure S2 in Supplementary Materials).

**Table 3:** Multivariable linear regression models for associations between cardiovascular risk factors and CAC progression stratified by sex with the use of < 30 kg/m^2^ (for BMI) and < 125 mmHg (for systolic BP) as references

Risk variable	Men		Women	
	β	*P* value	β	*P* value
Age	0.14	< 0.0001	0.022	0.389
White	0.056	0.077	0.05	0.189
BMI ≥ 30 kg/m^2^	0.13	0.004	0.045	0.196
Total Cholesterol	0.04	0.756	-0.037	0.654
Triglycerides	0.019	0.649	-0.043	0.155
LDL-C	0.055	0.519	0.064	0.444
Fasting glucose	0.074	0.202	-0.001	0.986
Systolic BP ≥ 125 mmHg	0.038	0.389	0.093	0.033
Smoking	0.085	0.03	0.12	0.007

Regression coefficients were calculated using robust standard errors

Model: Contained age, race, BMI, total cholesterol, triglycerides, systolic BP, LDL-C, fasting glucose, and smoking

BMI, body mass index;

BP, blood pressure;

CAC, coronary artery calcification;

LDL-C, low-density lipoprotein cholesterol

Similar associations were observed for waist circumference (Figure S3-S4, and Table S2 in Supplementary Materials). In an exploratory analysis, a model was fitted to examine the associations of 15-year change (year 0 to year 15) in BMI and systolic BP with CAC progression (Figure S5 and Table S3 in Supplementary Materials). 15-year change in systolic BP in women and 15-year change in BMI in men were still associated with CAC progression.

### Sensitivity analysis

After additional adjustment for diastolic BP, the results remained largely unchanged (Figure S6 and Table S4 in Supplementary Materials). In terms of incident CAC, the results rendered largely similar (Table S5 in Supplementary Materials).

## Discussion

In this population-based cohort study, we observed significant sex differences in the association between cardiovascular risk factor and CAC progression among participants with CAC = 0. BMI (and/or waist circumference) was associated with CAC progression only in men. In women, the associations between systolic BP and CAC progression followed a nonlinear relationship.

Previous studies have shown that BMI, systolic BP, and smoking are established risk factors for CAC progression among participants with CAC = 0. In the Multi-Ethnic Study of Atherosclerosis cohort involving 5756 individuals without baseline cardiovascular disease, Kronmal et al. found that BMI, hypertension, and smoking were associated with CAC incidence [9]. In the Atherosclerosis Risk in Communities study, Wang et al. observed that lower systolic BP was associated with a greater probability of CAC = 0 at older age [11]. However, data on sex differences in the associations between these cardiovascular risk factors and CAC progression are lacking. Our study confirmed and extended prior studies by examining whether cardiovascular risk factors were associated with CAC progression by sexes. Our observations suggested that among participants with CAC = 0, BMI (and/or waist circumference) was associated with CAC progression only in men, and the associations between systolic BP and CAC progression in women followed a nonlinear relationship. In addition, we also observed a nonlinear relationship between systolic BP and CAC progression in the entire population; it may be the reason why the interaction between sexes in systolic BP and CAC progression in the multivariable linear regression model did not reach statistical significance.

Compared to men, hypertension contributes to more cardiovascular disease events in women [22]. There is also evidence that the presence of any CAC is linked to CHD events and death [1, 3]. Consistent with previous results, our study found systolic BP was associated with CAC progression only in women and suggested that systolic BP in women may contribute to more cardiovascular disease events through accelerating CAC progression. Several mechanisms may account for the sex differences in the association between systolic BP and CAC progression observed in our study. First, sex specificity regarding the optimal range of systolic BP may remain unrecognized [23]. Prior studies have shown that basal systolic BP values existed within a lower normal range for women than for men [24], and cardiovascular disease risk was associated with elevations from lower systolic BP ranges in women compared with men [23]. Therefore, exposures leading to systolic BP elevation above sex-specific normal ranges may also elevate cardiovascular disease risk in a sex-specific manner. Potential mechanisms may also include differences in vascular anatomy and physiology [25], hormonal or non-hormonal biological variation that has yet to be identified [26], or a greater proportion of arterial stiffness in women than men [27]. As there is no evidence that the BP threshold for initiating drug treatment or the treatment target for lowering BP differs for women versus men, current hypertension guidelines have few special recommendations for management of hypertension in women [28]. Our findings support intensive treatment to lower systolic BP in women with CAC = 0 and provide insights into its potential added benefits. Randomized clinical trials with long-term follow-up periods are needed to confirm whether women with CAC = 0 can benefit from intensive systolic BP reduction. We also observed that BMI (and/or waist circumference) was associated with CAC progression in men, this association may be explained by sex-related differences in the adipocytes regarding their inherent metabolic biology [29]. Alternatively, sex-specific body fat distribution may also contribute to CAC progression, visceral fat volume in men is higher than that seen in women [30, 31].

There are some limitations to our study. First, this was a prospective observational study and cannot establish causality, and residual confounders caused by unknown factors cannot be ruled out. Second, we investigated the association of risk factors with CAC progression stratified by sex rather than cardiovascular events, which should be interpreted with caution, but previous studies have shown that CAC progression itself is a predictor of cardiovascular events [2, 4]. Third, some covariates were based on self-reports. Fourth, participants had to survive until CARDIA year 20 exam and participate in CT scans in years 15 and 20 to be included in the study, which may introduce bias. Finally, the Agatston CAC score carries some degree of measurement error and variability, particularly in the low score range, which may influence the results.

## Conclusion

In conclusion, in our study, we found that systolic BP in women and BMI (and/or waist circumference) in men may have different contributions to CAC progression between the sexes among participants with CAC = 0. Our study provides evidence that understanding sex differences in cardiovascular risk factors is essential for implementing targeted interventions to prevent CAC progression.

## Supplementary Information

Supplementary Material 1

## Data Availability

Not available.
