# Supplementary material for: Sex differences in the association between cardiovascular risk factors and coronary artery calcification progression among individuals without coronary artery calcium
Source: Biol Sex Differ. 2025 Dec 7;17:6. doi: 10.1186/s13293-025-00802-8 (PMC12798121; doi:10.1186/s13293-025-00802-8)
Supplement: Supplementary file 1 — Supplementary Material 1 [file 13293_2025_802_MOESM1_ESM.docx]

**Sex differences in the association between cardiovascular risk factors and coronary artery calcification progression among individuals without coronary artery calcium**

**Supplementary Materials**

Figure S1: Flowchart of participants selection (N=1815)


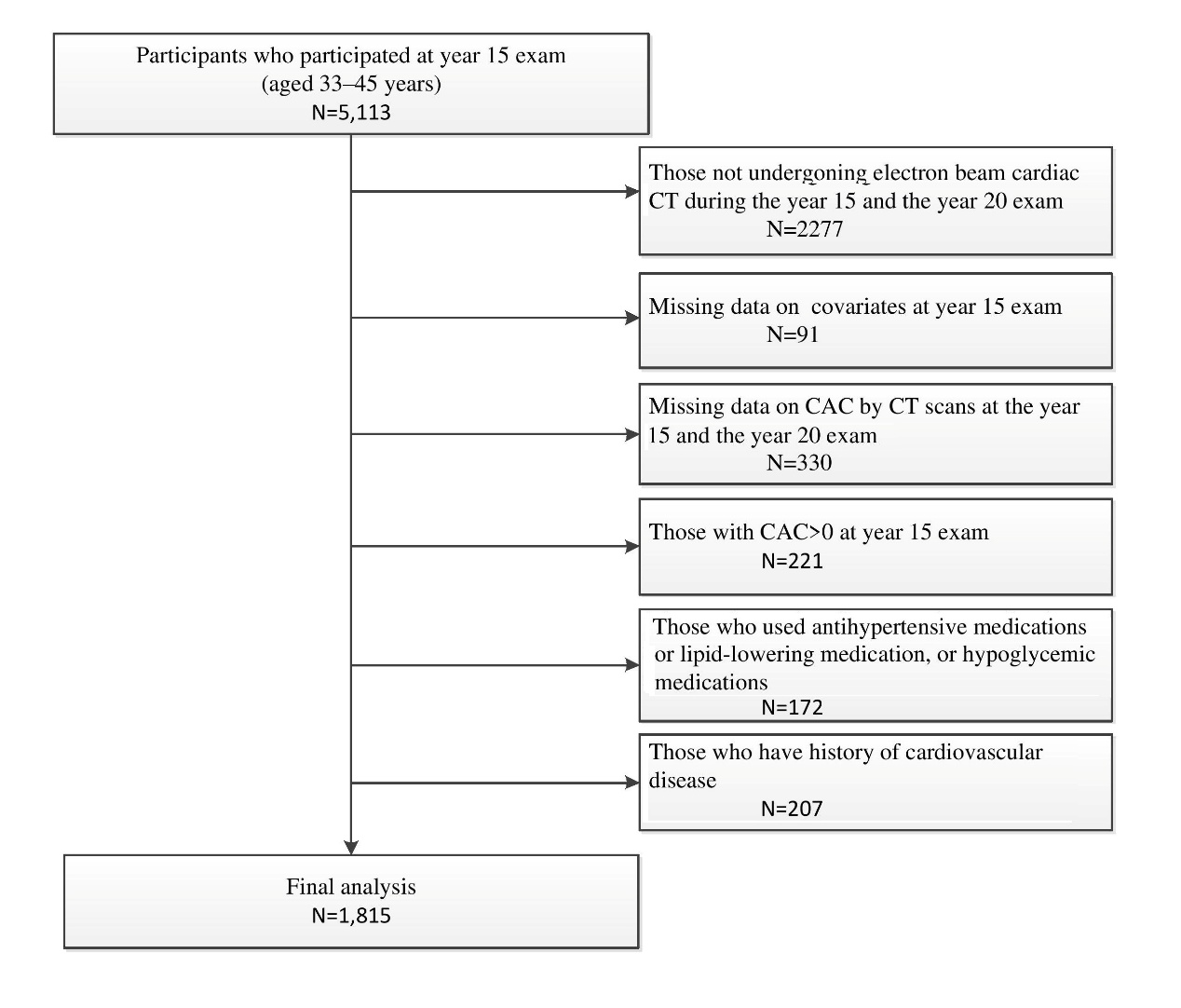


BMI, body mass index; BP, blood pressure

**CAC, coronary artery calcification**

CARDIA, Coronary Artery Risk Development in Young Adults;

**CT, computed tomography**

LDL-C, low-density lipoprotein cholesterol

Figure S2: Multivariable adjusted spline of association between cardiovascular risk factors and CAC progression in the entire population


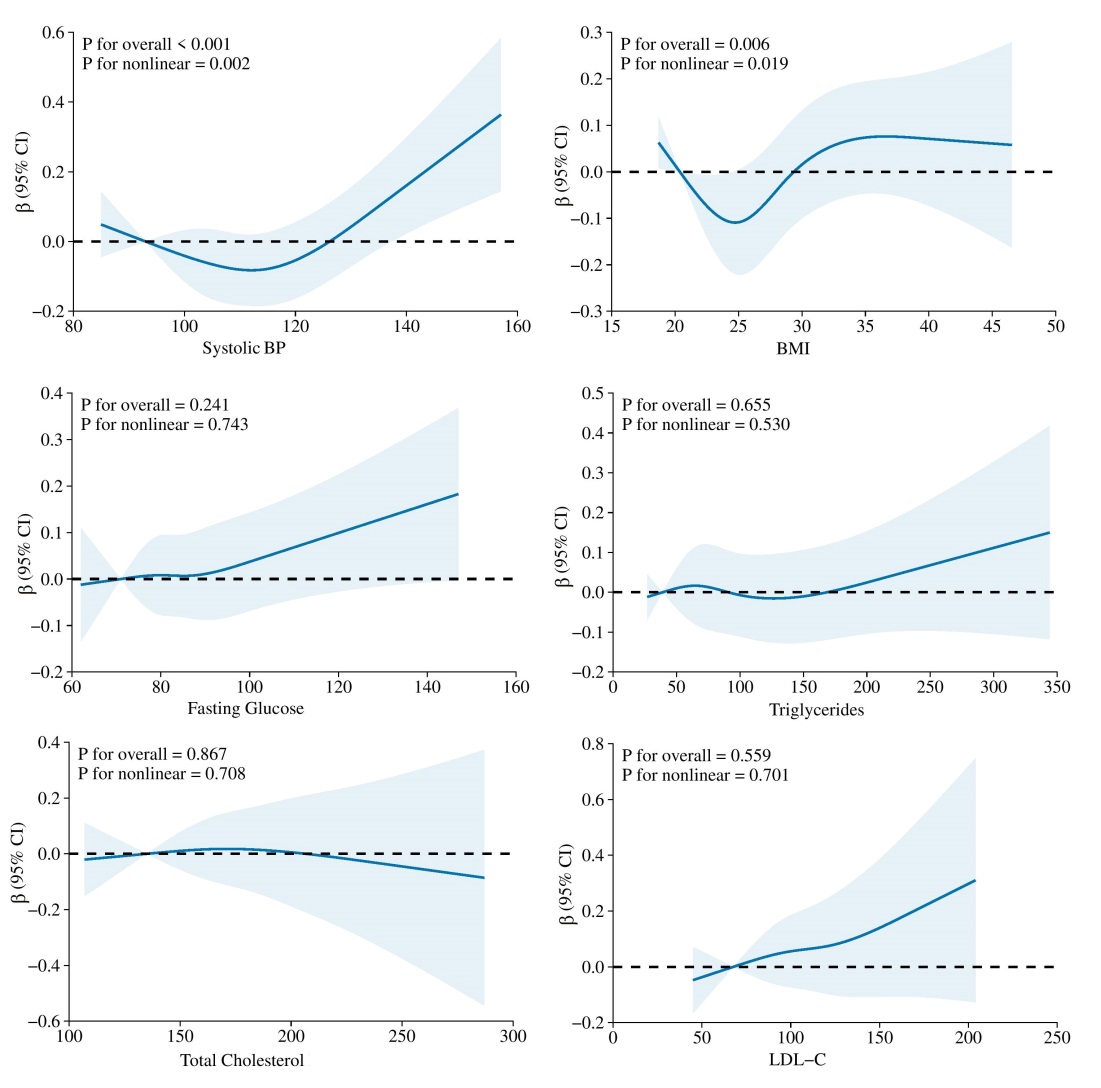


Graphs show β for CAC progression according to cardiovascular risk factors.

Model: Contained age, race, BMI, total cholesterol, triglycerides, systolic BP, LDL-C, fasting glucose, and smoking.

The model was conducted with 4 knots at the 5th, 35th, 65th, 95th percentiles (reference is the 5th percentile). Solid lines indicate β, and shadow shape indicates 95% CIs.

BMI, body mass index

BP, blood pressure

**CAC, coronary artery calcification**

CI, confidence interval

LDL-C, low-density lipoprotein cholesterol

Figure S3: Multivariable adjusted spline of association between cardiovascular risk factors and CAC progression stratified by sex replacing BMI with waist circumference


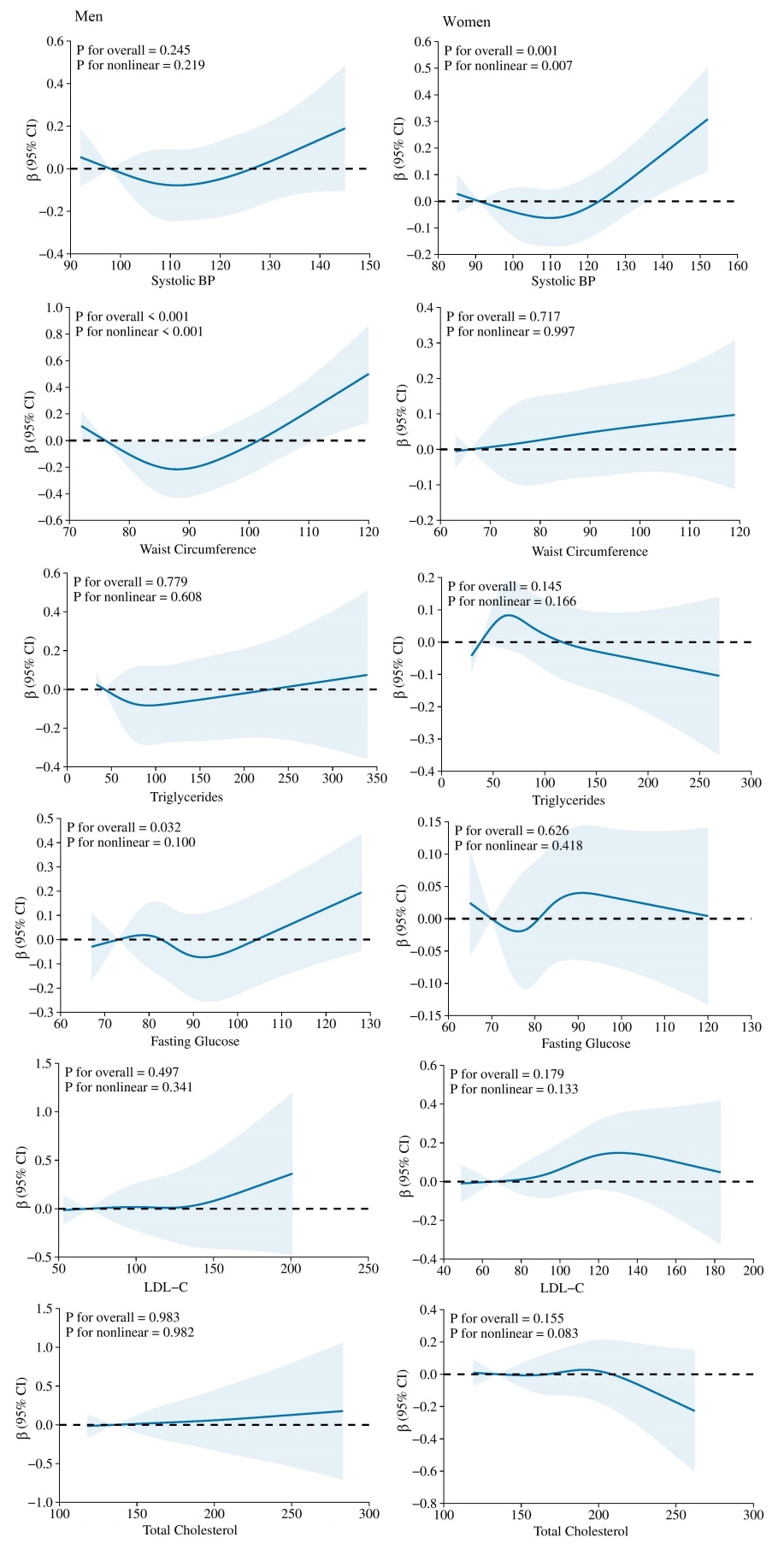


Graphs show β for CAC progression according to cardiovascular risk factors.

Model: Contained age, race, waist circumference, total cholesterol, triglycerides, systolic BP, LDL-C, fasting glucose, and smoking.

The model was conducted with 4 knots at the 5th, 35th, 65th, 95th percentiles (reference is the 5th percentile). Solid lines indicate β, and shadow shape indicates 95% CIs.

BMI, body mass index;

BP, blood pressure;

CAC, coronary artery calcification;

LDL-C, low-density lipoprotein cholesterol.

Figure S4: Multivariable adjusted spline of association between cardiovascular risk factors and CAC progression stratified by sex replacing BMI with waist circumference in the entire population


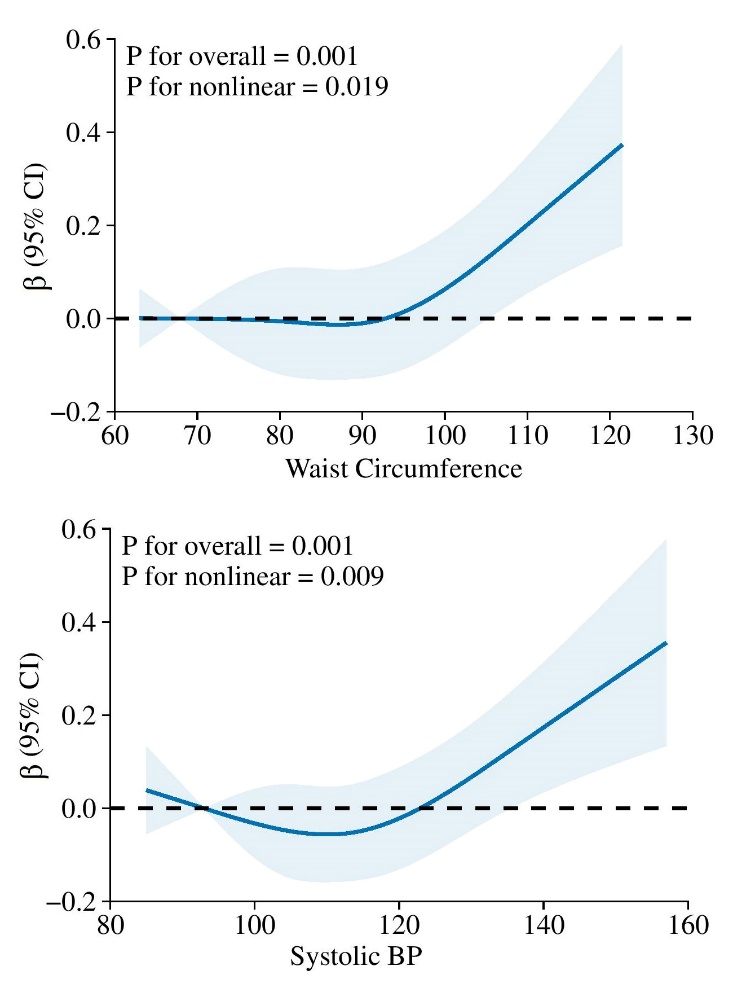


Graphs show β for CAC progression according to cardiovascular risk factors.

Model: Contained age, race, waist circumference, total cholesterol, triglycerides, systolic BP, LDL-C, fasting glucose, and smoking.

The model was conducted with 4 knots at the 5th, 35th, 65th, 95th percentiles (reference is the 5th percentile). Solid lines indicate β, and shadow shape indicates 95% CIs.

BMI, body mass index;

BP, blood pressure;

CAC, coronary artery calcification;

LDL-C, low-density lipoprotein cholesterol.

Figure S5: Multivariable adjusted spline of associations of 15-year change (year 0 to year 15) in BMI and systolic BP with CAC progression stratified by sex^a^


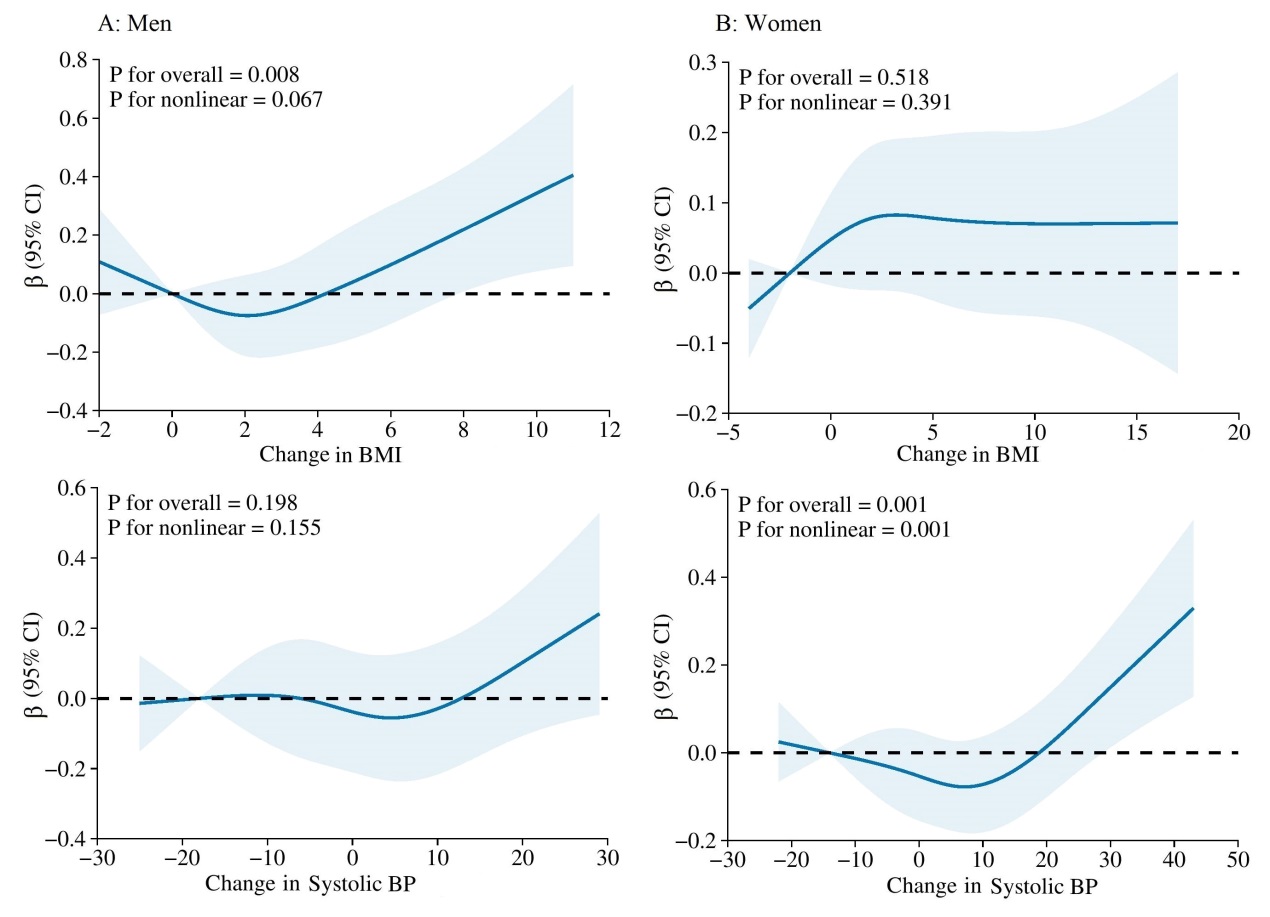


^a^Calculated as year 15 − year 0

Graphs show β for CAC progression according to cardiovascular risk factors.

Model: Contained age, race, smoking at year 15, and 15-year change (year 0 to year 15) in cardiovascular risk factors (BMI, total cholesterol, triglycerides, systolic BP, LDL-C, and fasting glucose)

The model was conducted with 4 knots at the 5th, 35th, 65th, 95th percentiles (reference is the 5th percentile). Solid lines indicate β, and shadow shape indicates 95% CIs.

BMI, body mass index

BP, blood pressure

**CAC, coronary artery calcification**

CI, confidence interval

LDL-C, low-density lipoprotein cholesterol

Figure S6: Multivariable adjusted spline of association between cardiovascular risk factors and CAC progression stratified by sex additionally adjusted for diastolic BP


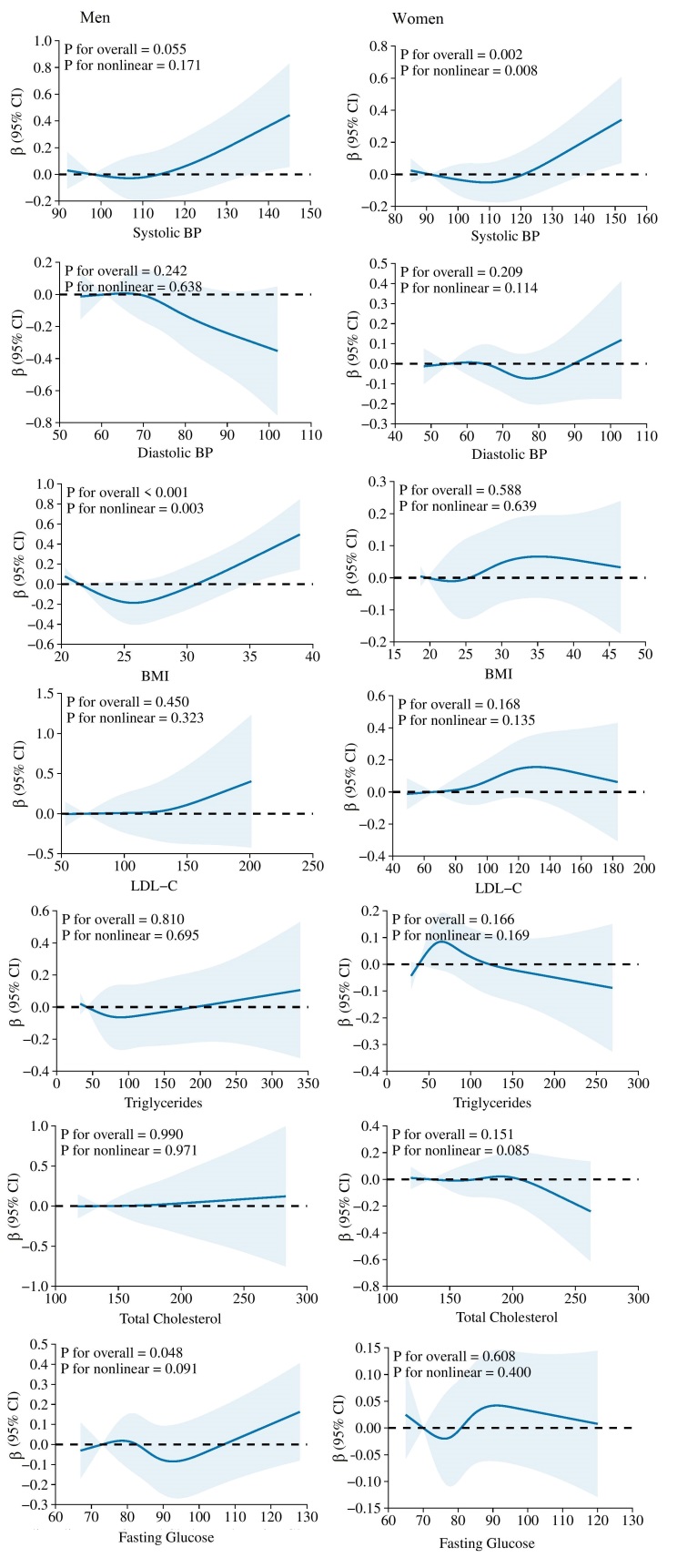


Graphs show β for CAC progression according to cardiovascular risk factors.

Model: Contained age, race, waist circumference, total cholesterol, triglycerides, systolic BP, diastolic BP, LDL-C, fasting glucose, and smoking.

The model was conducted with 4 knots at the 5th, 35th, 65th, 95th percentiles (reference is the 5th percentile). Solid lines indicate β, and shadow shape indicates 95% CIs.

BMI, body mass index;

BP, blood pressure;

CAC, coronary artery calcification;

LDL-C, low-density lipoprotein cholesterol.

Table S1: Associations between cardiovascular risk factors and CAC progression stratified by sex

| Risk variable | Men | | Women | |
| --- | --- | --- | --- | --- |
|  | β | *P* value | β | *P* value |
| (A) Univariate models |  |  |  |  |
| Age | 0.16 | <0.0001 | 0.035 | 0.189 |
| White | 0.054 | 0.12 | -0.001 | 0.976 |
| BMI | 0.15 | <0.001 | 0.052 | 0.087 |
| Total Cholesterol | 0.12 | 0.001 | 0.021 | 0.378 |
| Triglycerides | 0.11 | 0.007 | -0.003 | 0.885 |
| LDL-C | 0.11 | 0.004 | 0.04 | 0.15 |
| Fasting glucose | 0.12 | 0.021 | 0.017 | 0.455 |
| Systolic BP | 0.087 | 0.037 | 0.094 | 0.055 |
| Smoking | 0.053 | 0.196 | 0.11 | 0.01 |
| (B) Multivariable models^a^ |  |  |  |  |
| BMI | 0.16 | <0.001 | 0.057 | 0.077 |
| Total Cholesterol | 0.11 | 0.002 | 0.02 | 0.425 |
| Triglycerides | 0.096 | 0.017 | -0.003 | 0.874 |
| LDL-C | 0.11 | 0.004 | 0.039 | 0.164 |
| Fasting glucose | 0.11 | 0.035 | 0.014 | 0.52 |
| Systolic BP | 0.088 | 0.041 | 0.1 | 0.051 |
| Smoking | 0.06 | 0.137 | 0.11 | 0.01 |

Regression coefficients were calculated using robust standard errors.

a Adjusted for age and race

BMI, body mass index;

BP, blood pressure;

CAC, coronary artery calcification;

LDL-C, low-density lipoprotein cholesterol

Table S2: Multivariable linear regression models for associations between cardiovascular risk factors and CAC progression stratified by sex replacing BMI with waist circumference.

| Risk variable | P for interaction | Men | | Women | |
| --- | --- | --- | --- | --- | --- |
|  |  | β | *P* value | β | *P* value |
| Age |  | 0.14 | <0.0001 | 0.02 | 0.491 |
| White |  | 0.04 | 0.246 | 0.061 | 0.093 |
| Waist circumference | 0.003 | 0.12 | 0.008 | 0.045 | 0.268 |
| Total Cholesterol | 0.005 | 0.041 | 0.701 | -0.041 | 0.643 |
| Triglycerides | <0.001 | 0.015 | 0.755 | -0.05 | 0.119 |
| LDL-C | 0.018 | 0.052 | 0.623 | 0.071 | 0.444 |
| Fasting glucose | 0.046 | 0.074 | 0.282 | -0.002 | 0.912 |
| Systolic BP | 0.403 | 0.039 | 0.37 | 0.091 | 0.077 |
| Smoking | 0.999 | 0.08 | 0.039 | 0.11 | 0.009 |

Regression coefficients were calculated using robust standard errors.

Model: Contained age, race, waist circumference, total cholesterol, triglycerides, systolic BP, LDL-C, fasting glucose, and smoking.

BMI, body mass index;

BP, blood pressure;

CAC, coronary artery calcification;

LDL-C, low-density lipoprotein cholesterol.

Table S3: Multivariable linear regression models for associations of 15-year change (year 0 to year 15) in BMI and systolic BP with CAC progression stratified by sex^a^

| Risk variable | P for interaction | Men | | Women | |
| --- | --- | --- | --- | --- | --- |
|  |  | β | *P* value | β | *P* value |
| 15-year Change in BMI level | 0.139 | 0.11 | 0.025 | 0.024 | 0.46 |
| 15-year Change in Systolic BP level | 0.653 | 0.035 | 0.402 | 0.07 | 0.174 |

^a^Calculated as year 15 − year 0

Regression coefficients were calculated using robust standard errors.

Model: Contained age, race, smoking at year 15, and 15-year change (year 0 to year 15) in cardiovascular risk factors (BMI, total cholesterol, triglycerides, systolic BP, LDL-C, and fasting glucose)

BMI, body mass index

BP, blood pressure

**CAC, coronary artery calcification**

CI, confidence interval

LDL-C, low-density lipoprotein cholesterol

Table S4: Multivariable linear regression models for associations between cardiovascular risk factors and CAC progression stratified by sex additionally adjusted for diastolic BP

| Risk variable | P for interaction | Men | | Women | |
| --- | --- | --- | --- | --- | --- |
|  |  | β | *P* value | β | *P* value |
| Age |  | 0.15 | <0.0001 | 0.02 | 0.497 |
| White |  | 0.054 | 0.115 | 0.061 | 0.098 |
| BMI | 0.002 | 0.13 | 0.003 | 0.038 | 0.285 |
| Total Cholesterol | 0.006 | 0.027 | 0.802 | -0.047 | 0.595 |
| Triglycerides | 0.001 | 0.022 | 0.634 | -0.045 | 0.135 |
| LDL-C | 0.018 | 0.066 | 0.535 | 0.078 | 0.391 |
| Fasting glucose | 0.051 | 0.064 | 0.361 | -0.001 | 0.992 |
| Systolic BP | 0.39 | 0.1 | 0.078 | 0.11 | 0.136 |
| Diastolic BP | 0.96 | -0.093 | 0.05 | -0.021 | 0.719 |
| Smoking | 0.951 | 0.081 | 0.033 | 0.11 | 0.008 |

Regression coefficients were calculated using robust standard errors.

Model: Contained age, race, waist circumference, total cholesterol, triglycerides, systolic BP, diastolic BP, LDL-C, fasting glucose, and smoking.

BMI, body mass index;

BP, blood pressure;

CAC, coronary artery calcification;

LDL-C, low-density lipoprotein cholesterol.

Table S5: Associations between cardiovascular risk factors and incident CAC stratified by sex

| Risk variable | Men | | Women | |
| --- | --- | --- | --- | --- |
|  | OR (95% CI) | *P* value | OR (95% CI) | *P* value |
| Age | 1.11 (1.04-1.18) | 0.001 | 1.03 (0.96-1.12) | 0.406 |
| White | 1.43 (0.91-2.23) | 0.119 | 1.52 (0.82-2.83) | 0.181 |
| BMI, per 1 kg/m^2^ | 1.11 (1.06-1.17) | <0.0001 | 1.03 (0.99-1.08) | 0.196 |
| Total Cholesterol, per 1 mg/dL | 0.99 (0.98-1.02) | 0.896 | 0.99 (0.97-1.01) | 0.286 |
| Triglycerides, per 1 mg/dL | 1 (0.99-1.01) | 0.731 | 1 (0.99-1.01) | 0.656 |
| LDL-C, per 1 mg/dL | 1.01 (0.99-1.03) | 0.279 | 1.02 (0.99-1.04) | 0.122 |
| Fasting glucose, per 1 mg/dL | 1.01 (0.99-1.02) | 0.407 | 0.99 (0.98-1.02) | 0.895 |
| Systolic BP, per 1 mmHg | 1.01 (0.99-1.02) | 0.514 | 1.02 (1-1.04) | 0.032 |
| Smoking | 1.47 (0.89-2.42) | 0.134 | 2.18 (1.22-3.91) | 0.009 |

Model: Contained age, race, BMI, total cholesterol, triglycerides, systolic BP, LDL-C, fasting glucose, and smoking.

BMI, body mass index

BP, blood pressure

**CAC, coronary artery calcification**

CI, confidence interval

LDL-C, low-density lipoprotein cholesterol

OR, odds ratio
